# Growth patterns in early juvenile idiopathic arthritis: Results from the Childhood Arthritis Prospective Study (CAPS)

**DOI:** 10.1016/j.semarthrit.2017.11.002

**Published:** 2018-08

**Authors:** Flora McErlane, Roberto Carrasco, Lianne Kearsley-Fleet, Eileen M. Baildam, Lucy R. Wedderburn, Helen E. Foster, Yiannis Ioannou, S.E. Alice Chieng, Joyce E. Davidson, Wendy Thomson, Kimme L. Hyrich

**Affiliations:** a Paediatric Rheumatology, Great North Children’s Hospital, Newcastle Hospitals NHS Trust, Newcastle upon Tyne, UK; b Arthritis Research UK Centre for Epidemiology, Centre for Musculoskeletal Research, Division of Musculoskeletal & Dermatological Sciences. Faculty of Biology, Medicine and Health, The University of Manchester, Manchester, UK; c Paediatric Rheumatology, Alder Hey Children’s Hospital, Liverpool, UK; d Arthritis Research UK Centre for Adolescent Rheumatology, Infection, Inflammation and Rheumatology Section, UCL GOS Institute of Child Health, London, UK; e Arthritis Research UK Centre for Adolescent Rheumatology, Division of Medicine, University College London (UCL), London, UK; f Rheumatology, Royal Manchester Children’s Hospital, Manchester, UK; g Rheumatology, Institute Cellular Medicine, Newcastle University, Newcastle upon Tyne, UK; h Paediatric Rheumatology, Royal Hospital for Children, Glasgow, UK; i Paediatric Rheumatology, Royal Hospital for Sick Children, Edinburgh, UK; j Arthritis Research UK Center for Genetics and Genomics, Centre for Musculoskeletal Research, Division of Musculoskeletal & Dermatological Sciences. Faculty of Biology, Medicine and Health, The University of Manchester, Manchester, UK; k NIHR Manchester Musculoskeletal Biomedical Research Unit, Central Manchester NHS Foundation Trust, Manchester Academic Health Science Centre, Manchester, UK

**Keywords:** Juvenile idiopathic arthritis, Growth restriction, Height velocity, functional disability, Steroids

## Abstract

**Objectives:**

To investigate early vertical growth patterns and factors associated with poor growth in a modern inception cohort of UK children with juvenile idiopathic arthritis (JIA) using data from the Childhood Arthritis Prospective Study (CAPS).

**Methods:**

A study period of 3 years was chosen. Children included in this analysis had a physician diagnosis of JIA and had height measurements available at both baseline and at 3-years of follow-up. Height is presented as *z*-scores calculated using World Health Organisation growth standards for age and gender. Growth over the 3-year period was assessed using change in *z*-score and height velocity. Univariable and multivariable linear regressions were used to identify factors associated with height *z*-score at baseline and change of height *z*-score at 3 years.

**Results:**

568 patients were included; 65% female, median baseline age 7.4 years [interquartile range (IQR) 3.6, 11.2], median symptom duration at presentation 5.5 months [IQR 3.1, 11.6]. Height *z*-score decreased significantly from baseline to 3 years (*p* ≤ 0.0001); baseline median height *z*-score was −0.02 (IQR −0.71, 0.61), decreasing to −0.47 (IQR −1.12, 0.24) at 3 years. Growth restriction, defined as change of height *z*-score ≤−0.5, was observed in 39% of patients. At 3 years, higher baseline height *z*-score was the strongest predictor for a negative change in height *z*-score [−0.3 per unit of baseline height *z*-score (95% CI: −0.36, −0.24), *p* < 0.0001].

**Conclusions:**

Although overall height at 3 years after initial presentation to rheumatology is within the population norm, as a cohort, children with JIA experience a reduction of growth in height over the first 3 years of disease. Late presentation to paediatric rheumatology services is associated with lower height at presentation. However, patients with the lowest height *z* scores at presentation were also the most likely to see an improvement at 3 years. The impact of JIA on growth patterns is important to children and families and this study provides useful new data to support informed clinical care.

**Key messages:** 

• The study identified that growth restriction in JIA can occur early after disease onset. • Growth restriction was observed in 39% of patients. • Decrease in height *z*-score occurred across all JIA subtypes and was greatest in sJIA and PsJIA.

## Introduction

Inflammatory arthritis is one of the most common chronic inflammatory illnesses in childhood. It has been estimated that approximately 1:10,000 children will develop an inflammatory arthritis each year [1] with the majority subsequently diagnosed with juvenile idiopathic arthritis (JIA).

Growth disturbance is an important complication of JIA, with significant implications for both physical and psychosocial health. Although initially reversible, long-standing growth impairment results in irreversible short stature and altered adult body composition. It is a significant concern for the families of young children with JIA and an additional challenge for older children and adolescents coping with the impact of chronic illness [2].

Understanding the prevalence of short stature in JIA has traditionally been challenging, with estimates ranging from 1% to 17% [3]. Previous studies have defined juvenile arthritis according to different classification criteria and have included different subtypes of disease [4], [5], [6], using retrospective or cross-sectional study designs with variable lengths of follow-up, even within the same study. This risks the introduction of selection bias towards those children with the most severe disease requiring long-term rheumatological follow-up.

In recent years, biologic therapies such as etanercept and tocilizumab have been associated with improvements in vertical growth [7]. As a consequence, studies predating the widespread use of methotrexate or biologics may no longer be relevant to current populations of children and young people with JIA. More recent studies suggest that growth impairment persists in around 10% patients [8], despite intensification of treatment regimens and the advent of biologic therapies [7], [9], [10], [11].

The reasons for poor growth in JIA are multifactorial and may relate to the degree of systemic inflammation, corticosteroid use [12] or nutrition [13]; appetite may be impaired as a consequence of chronic inflammatory disease or as a side-effect of drugs with gastrointestinal toxicity such as methotrexate (MTX). Protein/energy malnutrition is thought to occur in children with JIA [14] and has been found to correlate with disease severity [15]. The extent of growth failure may vary with the ILAR subtype and is well described in children with polyarticular and systemic JIA [12], [16], [17]. In 2011, a review of 95 patients with oligoarticular JIA identified growth restriction in 36% patients [18]. Systemic corticosteroid use in JIA has been associated with a reduction in final adult height [12], [19], [20]. There is evidence that glucocorticoids interfere with the production or action of growth hormone and its mediators at different levels of the GH-insulin-like growth factor I axis [21].

The aim of this analysis was to investigate early growth patterns and factors associated with poor growth in a modern inception cohort of UK children and young people (CYP) with JIA over the first 3-years following diagnosis, using data from the Childhood Arthritis Prospective Study (CAPS).

## Materials and methods

### Study population

Children in this analysis were participants in CAPS, an ongoing inception cohort study launched in 2001 [22]. Children aged <16 years presenting to one of 7 paediatric and adolescent rheumatology referral centres across UK with a new diagnosis of inflammatory arthritis lasting for at least 2 weeks in at least one joint, are eligible to participate. The study was approved by the UK Northwest Multicentre Research Ethics Committee.

### Baseline data collection

Data are obtained through medical case note review, patient questionnaires and interview. Data collected include active and limited joint counts, 100-mm physician's global assessment (PGA) visual analogue scale (VAS), 100-mm pain VAS, and a 100-mm parent general evaluation (PGE) VAS, the child health assessment questionnaire (CHAQ). The physician assigns an International League Against Rheumatism (ILAR) subtype where appropriate. Additional demographic and health information data are provided by the families alongside completion of patient reported outcome questionnaires. Follow-up data are collected annually for the first 5 years following presentation and include all of the same information as collected at baseline. When children are discharged from paediatric rheumatology care, a study nurse will continue to collect follow-up data for a further 2 years.

### Measurement of height

The height in centimetres and weight in kilograms of all children are measured routinely during hospital clinic appointments as per local hospital protocol and recorded in the hospital case notes. For the purpose of this study, height measurements from the first paediatric rheumatology visit constituted baseline. Study nurses were advised to record the measurements which most closely corresponded to the study follow-up intervals, along with the date of measurement. Body mass index (BMI) was calculated subsequently.

### Analysis

A study period of 3 years was chosen to enable capture of definitive treatment data and early growth patterns. Children were included in this analysis if they had a diagnosis of JIA and had height measurements available at both baseline and at 3-years (± 3 months) of follow-up. Height and BMI are presented as *z*-scores, a standard score that indicates how many standard deviations an observation differs from the age and gender adjusted population median. Population data for the determination of *z*-scores were obtained from the World Health Organisation (WHO) website (http://www.who.int/childgrowth/en/) and the following calculation per child and height/BMI measurement was used: *[observed value-median value of reference population]/standard deviation (sd) of reference population].*

Height velocity was calculated using the following formula:

ΔHeight (cm)/Time (years)

Growth over the 3 years period was assessed using change in *z*-score and height velocity. As height *z*-score is a standardised measure, children would be expected to remain at the same height *z*-score over time, therefore any negative change in the *z*-score is considered a poor growth rate. We defined poor growth as a change of height *z*-score ≤−0.5 and severe growth restriction as height *z*-score change ≤−1 as previously described [7], [18]. Short stature was defined as a height *z*-score <−2 [18], [19]. Data are presented for the whole cohort and by ILAR category. As ILAR categories can change over time, we used the most recent ILAR category to allow children to “settle” into a category. Median annual height velocity over the 3-year period was plotted for each child and compared with WHO median height velocity standards. BMI classification was defined using the Extended International (IOTF) Body Mass Index Cut-Offs for Thinness, Overweight and Obesity in Children (http://www.iaso.org/resources/reports/newchildcutoffs).

Differences between baseline and 3 year height *z*-scores were compared using the Student’s paired *t* test. Univariable and multivariable linear regressions were performed to identify factors associated with (1) height *z*-score at baseline and (2) with the change of height *z*-score at 3 years. Co-variates in both models included baseline demographics [age at baseline, gender], patient reported outcome measures (PROMS) [CHAQ and pain VAS] and symptom duration at first presentation. In addition, model 1 included JADAS71 at baseline and the model 2 included change in JADAS71 from baseline to 3 years and treatment over the first 3 years [total time on oral/iv steroids in weeks, total number of intra-articular corticosteroid injections (IACI), DMARDS (yes/no) and biologic agents (yes/no)]. The results are presented as coefficients with 95% CIs. Multiple imputation was used to account for missing co-variate data, with 20 imputation sets generated. Analyses were performed in Stata version 13.1 (Statacorp 2003, College Station, TX, USA).

## Results

**Fig. 1:**
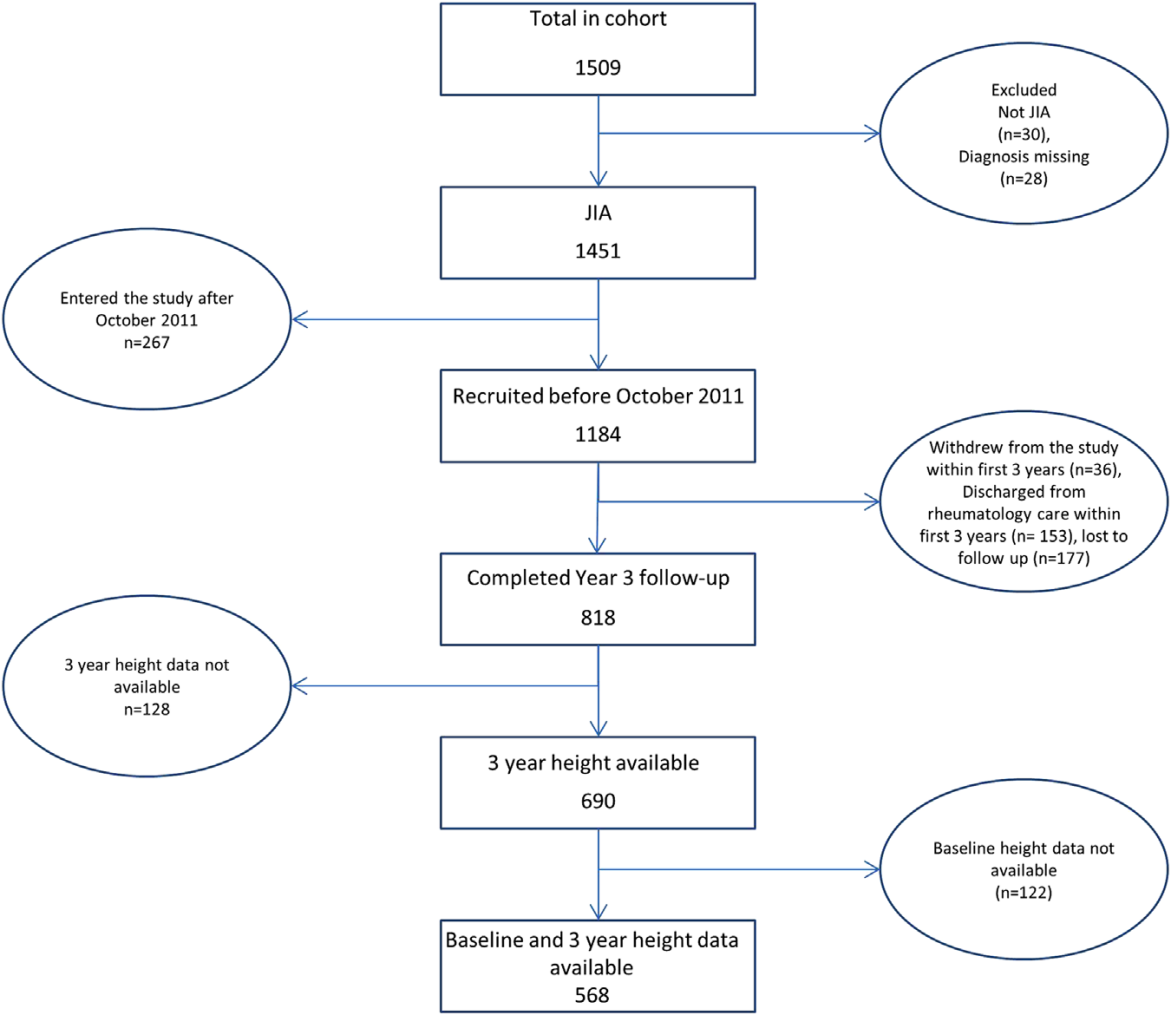
Patient selection flowchart.

Up to October 2014, CAPS had registered 1451 patients with JIA. Of these, 1184 had reached 3 years in the study of which 818 children had completed their 3-year follow-up. 690 of these children had height and weight recorded at 3 years but 122 had no height recorded at baseline, leaving 568 children for inclusion (Fig. 1). Compared to children who had reached 3 years in the study but did not have available height and weight data at both time points, those included in the analysis were slightly younger at presentation, had longer disease duration at baseline and were more likely to have a polyarticular disease course (Table 1).

**Table 1:** Baseline characteristics of the analysis cohort and a comparison with children excluded from the analysis

Baseline characteristics	Analysis cohort (*N* = 568)	Excluded cohort (*N* = 616)	*p* Value
Age at baseline (median, IQR)	7.4 (3.6, 11.2)	7.9 (3.5, 12.3)	0.03
Female (*n*, %)	372 (65)	394 (64)	0.4
Ethnicity, Caucasian (*n*, %)	518 (91)	545 (89)	0.2
Symptom duration at first presentation (median, IQR), months	5.5 (3.1, 11.6)	5.22 (2.5, 10.9)	0.04

ILAR subtype (*n*, %)			
Systemic arthritis	21 (4)	54 (9)	0.02
Persistent oligoarthritis	240 (42)	296 (48)	
Extended oligoarthritis	54 ( 9)	36 (6)	
RF(−)polyarthritis	139 (24)	116 (19)	
RF(+)polyarthritis	28 (5)	13 (2)	
Enthesitis-related arthritis	30 (5)	31 (5)	
Psoriatic arthritis	41 (7)	39 (6)	
Undifferentiated arthritis	14 (2)	31 (5)	

Active joint counts (median, IQR)	2 (1, 5)	2 (1, 5)	0.2
Limited joint counts (median, IQR)	1 (1, 3)	1 (0, 3)	0.04
PGE, mm (median, IQR)	22 (6, 50)	20.5 (4, 50)	0.5
PGA, mm (median, IQR)	28.5 (16, 50)	29 (15, 50)	0.9
JADAS 71	9.9 (6, 17)	10.4 (5.5, 16.8)	0.6
Δ JADAS71 from baseline to 3 years	−6.6 (−13, −2.7)	−6.9 (−13.2, −2.4)	0.4
CHAQ (median, IQR) (0–3)	0.75 (0.25, 1.37)	0.625 (0.12, 1.37)	0.09
Pain, mm (median, IQR)	30 (9, 60)	30 (7, 57)	0.4

All values are median [IQR (interquartile range)] or *n* (%). Groups were compared using non-parametric statistics.

### Treatment

Over the three years following first presentation, 359 (63%) children received treatment with DMARDS, primarily methotrexate, 121 (21%) also received biologic therapy, 254 (44%) received systemic steroids over a total median cumulative time of 4.1 weeks (IQR 0.4, 22) and 448 (78%) received IACIs with median total number of injections of 4 (IQR 2, 8)/patient (Table 2).

**Table 2:** Medication use between baseline and 3 years according to ILAR category

	Whole cohort	Systemic arthritis	Persistent oligoarthritis	Extended oligoarthritis	RF(−) polyarthritis	RF(+) polyarthritis	ERA	PsA	Undifferentiated
*N*	568	21	240	54	139	28	30	42	14
Oral/IV steroids (yes/no)	254 (44)	19 (90)	48 (20)	27 (50)	92 (66)	23 (82)	18 (60)	19 (45)	8 (57)
Median (IQR) weeks cumulative use/child^a^	4.1 (0.3, 22.1)	46.6 (23.6, 116.1)	0.7 (0.1, 8.7)	1.8 (0.1, 12.1)	4.6 (0.6, 23.7)	15.5 (0.9, 42.7)	4.6 (0.6, 12.8)	2.9 (0.1, 13)	3 (1, 37)
IACIs (yes/no)	446 (78)	7 (33)	200 (83)	51 (94)	103 (75)	21 (75)	23 (77)	35 (83)	6 (42)
Median (IQR) IACIs/child^b^	4 (2, 8)	5 (5, 14)	2 (1, 4)	5 (3, 10)	7 (3, 12)	8 (4, 14)	3 (2, 8)	6 (3, 9)	2.5 (1, 10)
DMARD (yes/no)	358 (63)	19 (90)	72 (30)	45 (83)	135 (97)	28 (100)	22 (73)	31 (74)	6 (42)
Biologics (yes/no)	121 (21)	7 (33)	12 (5)	13 (24)	52 (37)	9 (32)	16 (53)	10 (24)	2 (14)

Values are *n* (%) unless otherwise indicated.

a Includes patients treated with oral/iv steroid.

b Includes patients treated with Intra-articular corticosteroids injection (IACI).

### Anthropomorphic data

**Fig. 2:**
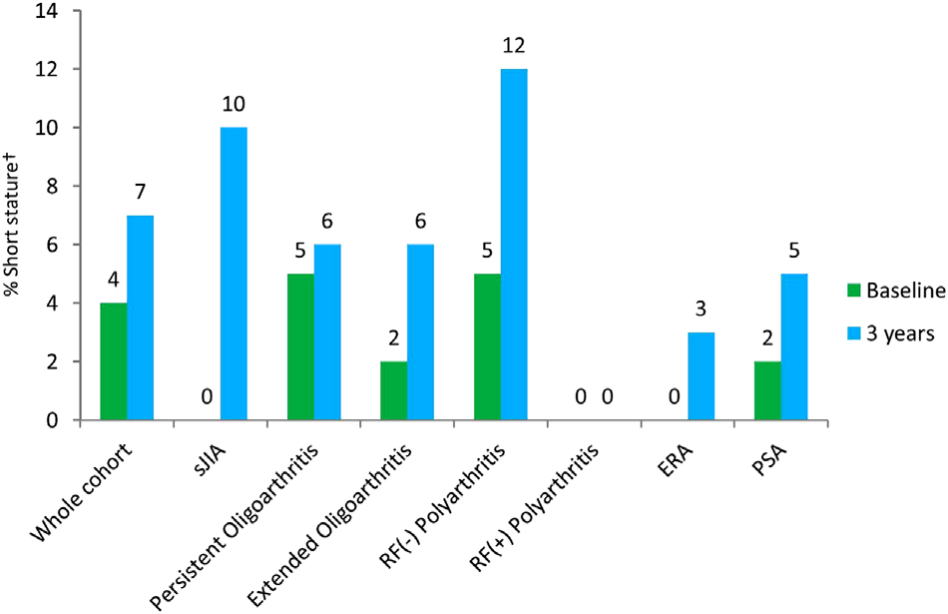
Short stature: presented for the whole cohort and by ILAR subtype. ^†^Short stature: height *z* score ≤ −2. Abbreviations: sJIA = systemic arthritis; RF = rheumatoid factor; ERA = enthesitis related arthritis; PSA = psoriatic arthritis.

**Fig. 3:**
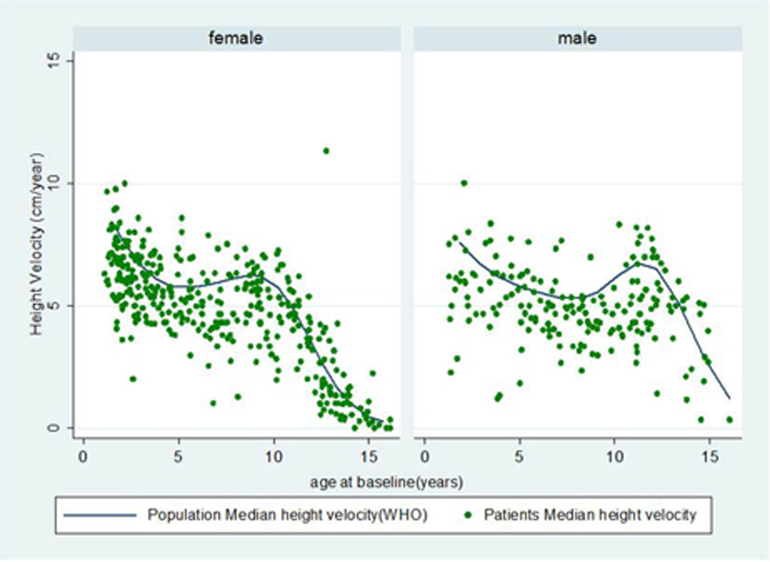
Average height velocity over 3 years of follow up by age at baseline (male and female). Median annual height velocity over the 3-year period plotted for each child and compared against age and gender matched reference population (WHO). Abbreviation: WHO = World Health Organisation.

**Fig. 4:**
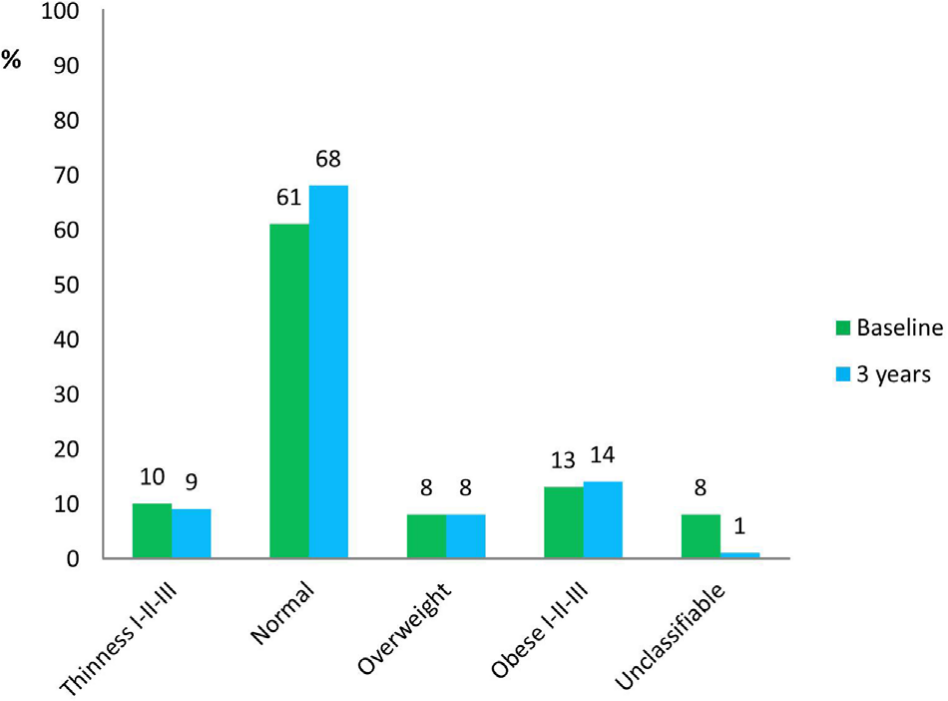
BMI classification at baseline and 3 years. BMI classification was defined using the Extended International (IOTF) Body Mass Index Cut-Offs for Thinness, Overweight and Obesity in Children. Abbreviations: BMI = body mass index; IOTF = International Obesity Task Force.

At presentation, median height *z*-score was −0.02 (IQR −0.71, 0.61) (Table 3). Twenty patients (4%) were classified as having short stature at baseline (Fig. 2). Median height *z*-score at 3 years decreased to −0.47 (IQR −1.12, 0.24), an overall median change of −0.31 (−0.80, 0.10). The 3-year median height *z*-score was significantly different from baseline height *z*-score (p ≤ 0.0001). Growth restriction was observed in 39% of patients (22% moderate and 17% severe growth restriction), and 42 (7%) had short stature after 3 years. Median height velocity was 5 cm/year (IQR 4, 6.2) with more than 70% of patients showing lower height velocity against age and gender matched reference population (Fig. 3). Decrease in height *z*-score occurred across all JIA subtypes and was greatest in systemic arthritis (sJIA) and psoriatic arthritis (PsA) with a change of −0.50 (IQR −0.97, −0.29) and −0.40 (IQR −1.02, 0.07), respectively. At baseline, median BMI *z*-score was slightly above the reference population [0.36 (IQR −0.35, 1.3)] (Supplementary Table). Overall, there was no appreciable change in median BMI *z*-score at 3 years [−0.11 (IQR −0.52, 0.39)], but there was an increase in children with sJIA [+0.33 (IQR −0.52, 0.66)] and moderate decrease in patients with extended oligoarthritis [−0.26 (IQR −0.65, 0.27)] and ERA [−0.32 (IQR −0.58, 0.04)]. One hundred and eighteen (23%) were overweight, obese or morbidly obese at first presentation and a similar proportion [123 (22%)] at year 3 (Fig. 4).

**Table 3:** Anthropomorphic data: presented for whole cohort and by ILAR category

					Growth restriction		
	*N*	Height *Z* score at baseline (median, IQR)	Height *Z* score at 3 years (median, IQR)	Δ Height *Z* score (median, IQR)	Moderate^a^ (*n*, %)	Severe^b^ (*n*, %)	Height velocity, cm/year (median, IQR)
Whole cohort	468	−0.02 (−0.71, 0.61)	−0.47 (−1.12, 0.25)	−0.31 (−0.80, 0.10)	223 (39)	99 (17)	5.1 (4, 6.2)
sJIA	21	0.03 (−0.25, 0.40)	−0.35 (−0.71, 0.02)	−0.50 (−0.96, −0.29)	11 (52)	5 (24)	4.2 (2, 5.2)
Persistent oligoarthritis	240	0.01 (−0.67, 0.67)	−0.50 (−1.01, 0.22)	−0.32 (−0.81, 0.08)	99 (41)	42 (18)	5.3 (4.2, 6.3)
Extended oligoarthritiss	54	−0.06 (−0.63, 0.78)	−0.58 (−1.25, 0.34)	−0.31 (−1.06, 0.16)	21 (39)	14 (26)	5.4 (4.4, 6.3)
RF(−)polyarthritis	139	−0.28 (−0.92, 0.46)	−0.53 (−1.27, 0.28)	−0.22 (−0.71, 0.12)	46 (33)	15 (11)	5.1 (4.1, 6)
RF(+)polyarthritis	28	0.18 (−0.53, 0.81)	−0.23 (−0.91, 0.54)	−0.27 (−1.03, 0.16)	12 (42.9)	8 (29)	4 (2.9, 5.7)
ERA	30	0.29 (−0.46, 0.86)	−0.27 (−0.73, 0.62)	−0.23 (−0.60 ,0.22)	10 (33)	3 (10)	5.1 (3.9, 6)
PSA	42	−0.25 (−0.76, 0.22)	−1.02 (−1.34, −0.03)	−0.40 (−1.02, 0.07)	20 (48)	11 (26)	4.4 (3.7, 5.1)

Height *z*-score data at baseline and changes after 3 years of follow up. Values are *n* (%) or median (IQR).

a Moderate growth restriction: change in height *Z* score ≤ −0.5

b Severe growth restriction: change in height *Z* score ≤−1.

### Factors associated with height *z*-score and height *z*-score changes

In multivariable analysis at baseline, higher CHAQ score, longer symptom duration and lower BMI *z*-score were significantly associated with lower height *z*-scores. Although higher JADAS-71 was significantly associated with lower height at baseline in the univariate analysis, no association was found in the multivariate model.

At 3 years, baseline height *z*-score was the strongest predictor for improved growth. For every unit decrease in baseline height *z*-score, a 0.3 unit increase in height *z*-score from baseline to 3 years was predicted [−0.3 per unit *z*-score (95% CI: −0.36, −0.24), *p* < 0.0001]. In addition, total time on oral or intravenous steroids during the 3-year period was significantly associated with decrease in height *z*-score from baseline to 3 years (Table 4).

**Table 4:** Factors associated with height *z*-score at baseline and with change in height *z*-score from baseline to 3 years

Co-variates at baseline	Univariable analysis at baseline, coefficient (95% CI)	Multivariable analysis at baseline, coefficient (95% CI)
BMI *z*-score (per unit)	0.1 (0.05, 0.2)^a^	0.1 (0.04, 0.2)⁎
Gender (female)	0.14 (−0.05, 0.3)	--
JADAS71 (per unit)	−0.01 (−0.02,−0.002)^a^	−0.001 (−0.01, 0.01)
CHAQ (per unit)	−0.2 (−0.4, −0.1)^a^	−0.2 (−0.4, −0.08)⁎
Pain VAS (mm)	−0.002 (−0.005, 0.002)	--
Age at baseline (year)	−0.005 (−0.03, 0.02)	--
Disease duration (per month)	−0.01 (−0.01, −0.003)^a^	−0.01 (−0.01, −0.002)⁎

Co-variates	3 years change, univariable analysis adjusted by medication,^b^ coefficient (95% CI)	3 years change, multivariable analysis adjusted by medication,^b^ coefficient (95% CI)

Baseline height *z*-score	−0.3 (−0.3, −0.2)^a^	−0.3 (−0.4, −0.2)⁎
BMI *z*-score change from baseline to 3 years	−0.1 (−0.2, −0.05)^a^	−0.1 (−0.15, −0.02)⁎
Gender (female)	−0.1 (−0.2, 0.03)	--
Disease duration (per month)	0.004 (−0.0001, 0.008)^a^	−0.002 (−0.006, 0.001)
Age at baseline (per year)	0.05 (0.03, 0.07)^a^	0.05 (0.04, 0.07)⁎
JADAS71 change from baseline to 3 years	−0.007 (−0.014, 0.0001)^a^	−0.004 (−0.01, 0.04)
CHAQ (per unit)	0.12 (0.03, 0.2)^a^	0.07 (−0.04, 0.2)
Pain VAS (mm)	0.002 (−0.001, 0.005)	--
Number IACI (per injection)	−0.012 (−0.02, 0.0003)	−0.012 (−0.02, 0.0004)
Oral/IV steroids (per week)	−0.001 (−0.004, 0.002)	−0.003 (−0.006, −0.0005)⁎
Ever had DMARD (yes/no)	0.04 (−0.1, 0.2)	−0.01 (−0.2, 0.1)
Ever had biologic (yes/no)	0.08 (−0.09, 0.2)	0.01 (−0.15, 0.18)

Univariable and multivariable linear regression for associations with baseline height *z*-score and with change in height *z*-score from baseline to 3 years.

a Variables with *p* ≤ 0.1 in the univariable analysis and therapy were included in the multivariable model.

b Medications were included as co-variates in the statistic model. -- Variable not included in the multivariable model.

⁎ *p* ≤ 0.05.

## Discussion

This is one of the largest longitudinal studies of vertical growth in all JIA subtypes, particularly focussing on changes to height in the first 3 years after diagnosis. Although height was within the normal range at presentation (−0.02), by capturing data systematically from first presentation to paediatric rheumatology we have identified that growth restriction can occur early after disease onset, both across the entire cohort and within each ILAR subtype. The simultaneous capture of multiple covariates has allowed us to explore the impact of disease activity and pharmacological interventions on growth over this early interval. The dataset was captured in a modern treatment era, and all children had access to biologic therapies. Previous analysis of this cohort has not shown that biologic use has increased significantly over the course of the study [23].

The greatest decrease in *z*-score was identified in CYP with sJIA (−0.5), with 24% experiencing a severe deceleration in vertical growth. Reduced growth velocity early in the course of sJIA has been reported previously [9], [24], [25] and is likely multifactorial. CYP with sJIA are often systemically unwell at presentation, necessitating treatment with high dose corticosteroids [24]. The impact of corticosteroids on growth velocity and final height is well-described [12], [20], [25], [26] but it remains unclear whether corticosteroids have an independent negative effect on growth [21] or simply represent a marker of disease severity [15], [27].

In our study, 90% of sJIA had systemic corticosteroids for a median duration of 46.6 weeks and it is likely that steroid exposure contributed to the reduced growth velocity. Total time on oral/IV corticosteroids over the 3 years was associated with growth restriction. We observed this association between corticosteroids and growth even after adjustment for disease activity, disease duration and DMARDs therapy. A previous study reported significant growth restriction in children with no exposure to corticosteroids [28], suggesting that growth restriction in sJIA is complex and may relate directly to the systemic inflammatory process.

High levels of IL-6 in children with sJIA may further reduce growth velocity. In 2001, Benedetti et al. [29] identified that chronic overproduction of IL-6 leads to decreased levels of IGF binding protein 3, suggesting that IL-6 contributes to the reduced growth velocity noted in chronic inflammatory states.

41% of CYP with persistent oligoarthritis experienced moderate growth restriction. Most children were treated with IACI rather than systemic therapies, with a trend for greater growth restriction among patients who had more IACIs (*p* = 0.06). Growth restriction has been reported previously in CYP with JIA treated with IACI [18]. Earlier use of DMARD in CYP requiring repeat IACI may reduce growth restriction in this group. Our data suggest that future studies exploring patterns of growth restriction and correlation with steroid prescribing patterns would add important information.

PsA was also associated with a significant decrease in *z*-score (−0.4), with 26% experiencing a severe deceleration in vertical growth. Low numbers make it difficult to draw firm conclusions about this subgroup but PsJIA is a multisystem disorder and the impact of chronic psoriasis on childhood growth is not well described. The extent of skin involvement was not recorded in CAPS but may be highly relevant. In particular, IL-6 is highly expressed in psoriatic skin lesions [30] and is thought to contribute to reduced growth velocity in chronic inflammation [29].

Delay in presentation to paediatric rheumatology was associated with lower height at presentation. Previous analysis of this same dataset identified that children with PsA have the longest mean time to first presentation to paediatric rheumatology [31], suggesting that delayed presentation may be an important risk factor for poor growth in PsA.

Delays in referral to paediatric rheumatology have previously been identified and may relate to delays in disease recognition [32], [33], [34], [35] or initial referral to specialties other than rheumatology [31]. It is encouraging that lower height at baseline predicts improvement in vertical growth over the first 3 years of follow up. Although this could be explained in part by regression to the mean, CYP with more pronounced growth restriction at presentation may demonstrate higher growth velocity with appropriate treatment [7], [9], [10], [36]. The association between delay from symptom onset to first presentation to paediatric rheumatology and height *z*-score change at 3 years was not significant.

A prominent predictor of lower baseline height was the functional disability at first presentation. This is in line with previous studies where functional class was identified as a factor related to growth impairment [19], [28]. There is also evidence from previous studies which correlate disease activity and poor growth [17], [18], [37]. However, in our analysis disease activity was not associated with height in multivariable analysis, possibly due to collinearity between disease activity and disability [38].

An increase in BMI from baseline to 3 years was also associated with poor growth in height in this cohort. It has been shown [39], [40] that BMI may be abnormal in chronic inflammatory disorders. A recent study of BMI among children with rheumatic diseases [39] describes that BMI trajectory reached a peak in the first 4 months of follow up and then returned to baseline values while height *z*-scores trajectories showed continued height deficits. We showed a similar trend with poor vertical growth but no significant overall change in median BMI *z*-score during the 3 years period.

Older age at presentation predicted improvement in vertical growth. Similarly, a study [7] in 191 etanercept-treated patients with JIA reported an association between improved growth rates in patients with ages between 11 and 14 years. This association may relate to the pubertal growth spurt. Conversely, another study [28] reported that linear growth observed in sJIA and polyarticular arthritis during the age of puberty was significantly slower than a group observed before puberty, suggesting that change in height during the puberty might be more sensitive to growth-affecting factors. Unfortunately, in our study, pubertal status data were unavailable. Although the median age at baseline was 7.4 years, it is reasonable to assume that a proportion of the study cohort (particularly the female participants) may have entered the pubertal growth spurt by three years after the initial presentation.

Previous publications have shown that control of disease activity is associated with growth improvement [17], [18], [36]. However, in our study, after adjustment for other factors, disease activity or change in disease activity, measured using the JADAS-71, was not associated with growth. As our measures of JADAS did not capture the entirety of disease activity over the follow-up period, these single measures in time may not accurately capture disease activity over the follow-up. In addition it is important to highlight that JADAS does not accurately capture overall disease activity in all subtypes, in particular sJIA, PsJIA and ERA [41]. Similarly, there is likely to be a strong correlation in many patients between disease activity and steroid use and disentangling the role either plays independent of the other is challenging. In some children, it is possible that using aggressive therapy with systemic steroids to achieve disease control may have outweighed any benefit of disease control. This would support the idea that disease control by aggressive therapeutic approach, but with a minimal use of systemic steroids, would help to prevent linear growth restriction in JIA patients [19], [25].

The anthropomorphic data captured in this study is real-life hospital data. Although all seven participating centres collect height and weight data according to strict hospital protocols, height measurements were not always taken at the same time of day or using the same measuring equipment. In addition, it was not possible to adjust for the impact of joint contractures or limb length discrepancy on the final height.

Missing anthropomorphic data constitute a further limitation. Although it is routine to capture height and weight at every paediatric rheumatology visit, in some cases height and weight data were either missing or were measured at a time too far from the 3 year study visit. Anthropomorphic data may be difficult to obtain in outreach or adult clinic settings and a proportion of missing data may be due to failure to attend clinical appointments. A further proportion of children were excluded as they did not complete 3 years within the study. The reasons for this were multifactorial and included children who had been discharged for remission prior to year 3. A further subset of adolescents would have been transferred to adult rheumatology services. We elected to exclude these patients rather than make any assumptions about their height based on earlier measurements. Another possible limitation is the no inclusion of co-morbidities in our analysis. Some of these co-morbidities, such as coeliac disease or inflammatory bowel disease could impact on the growth. An additional limitation is that CAPS do not collect pubertal status, markers of bone age and health. Some of this data has already been collected within other national cohort studies [42], [43], highlighting the importance of international collaborative studies in rare diseases such as JIA.

Although there were differences in ILAR distribution between those included and excluded, the overall differences were small suggesting these results can, in general, be applied to the entire cohort. Finally, the study did not have a record of parental height.

The analysis cohort has a longer disease duration at presentation than the cohort as a whole (*p* = 0.04) and a higher proportion of polyarticular disease patterns (*p* = 0.02). This suggests that the analysis cohort may include a higher proportion of children with more severe disease than routine clinical cohorts.

Identification of growth restriction in the first three years following presentation is important to children and young people with JIA. Growth delay has significant potential to impair both physical and psychosocial health and may impact on longer term educational and vocational outcomes. This study further highlights the need for early aggressive treatment regimes in routine clinical practice. Targeted early treatment pathways are likely to improve clinical outcomes in children with JIA, and this may include a reduction in early growth restriction [44].

## Conclusion

This large study has shown that although overall height at 3 years following presentation to rheumatology is within the population norm, as a cohort, children with JIA do experience an important loss in vertical growth over the first 3 years of disease. Continued follow-up of these children into adolescence will indicate if these early losses in vertical growth can be reversed prior to reaching final adult height. Future work should include collection of detailed growth data including pubertal status, markers of bone age and health and a detailed exploration of patterns of growth restriction in the different ILAR subtypes.

## Disclosure statement

K.L.H. has received honoraria from Pfizer and AbbVie for work unrelated to the present article. All other authors have declared no conflicts of interest.
