# Supplementary material for: Growth patterns in early juvenile idiopathic arthritis: Results from the Childhood Arthritis Prospective Study (CAPS)
Source: Semin Arthritis Rheum. 2018 Aug;48(1):53–60. doi: 10.1016/j.semarthrit.2017.11.002 (PMC6089842; doi:10.1016/j.semarthrit.2017.11.002)
Supplement: Supplementary file 1 — Supplementary material [file mmc1.docx]

Supplementary table. BMI data: presented for whole cohort and by ILAR Category

|  | N | BMI Z score at Baseline(Median, IQR) | BMI Z score at 3 years(Median, IQR) | Δ BMI Z score(Median, IQR) |
| --- | --- | --- | --- | --- |
| Whole cohort | 468 | 0.36 (-0.35, 1.30) | 0.34 (-0.40, 1.12) | -0.11 (-0.52, 0.39) |
| sJIA | 21 | 0.53 (-0.28, 1.71) | 1.06 (0.37, 2.1) | 0.33 (-0.52, 0.66) |
| Persistent Oligoarthritis | 240 | 0.36 (-0.34, 1.36) | 0.32 (-0.53, 1.0) | -0.17 (-0.59, 0.34) |
| Extended Oligoarthritiss | 54 | 0.65 (-0.34, 1.54) | 0.28 (-0.36, 1.12) | -0.26 (-0.65, 0.27) |
| RF(-)polyarthritis | 139 | 0.29 (-0.49, 1.10) | 0.39 (-0.32, 1.13) | 0.08 (-0.34, 0.60) |
| RF(+)polyarthritis | 28 | -0.03 (-0.88, 0.44) | -0.03 (-0.75, 0.32) | -0.09 (-0.35, 0.43) |
| ERA | 30 | 0.61 (-0.33, 1.08) | 0.07 (-0.32, 1.14 | -0.32 (-0.58, 0.04) |
| PSA | 42 | 0.49 (-0.23, 1.47) | 0.50 (-0.23, 1.28) | -0.16 (-0.54, 0.35) |
| BMI z-score data at baseline and changes after 3 years of follow up. Values are median (IQR) | | | | |
